# Lentinan dose dependence between immunoprophylaxis and promotion of the murine liver cancer

**DOI:** 10.18632/oncotarget.19808

**Published:** 2017-08-01

**Authors:** Ying Wang, Xue Han, Yan Dong Li, Yabing Wang, Shi Yang Zhao, Dong Jie Zhang, Yu Lu

**Affiliations:** ^1^ College of Food, Heilongjiang Bayi Agricultural University, Daqing 163319, PR China; ^2^ College of Biological Science and Engineering, Hebei University of Science and Technology, Shijiazhuang 050018, PR China; ^3^ Hebei Institute of Veterinary Drugs Control, Shijiazhuang 050000, PR China; ^4^ National Coarse Cereals Engineering Research Center, Daqing 163319, PR China; ^5^ Huabei Petroleum Administration Bureau, Huasheng Integrated Service, Tianjin 300000, PR China

**Keywords:** lentinan, dose dependence, liver cancer, immunoprophylaxis, promotion

## Abstract

Lentinan could exhibit significant biological activity favorable for human health and disease control such as the recovery of patients with liver cancer. In order to investigate the effect of lentinan dose dependence between immunoprophylaxis and promotion of cancer cell proliferation of the murine liver cancer, different concentrations of lentinan were prepared for the test *in vitro* (MTT assay) and *in vivo* (cumulative survival assay, spleen lymphocyte proliferation tests and peritoneal macrophage phagocytosis assays). New emerging proteins of the H22 cell incubated with lentinan was demonstrated by MS analysis and protein database searching. Lentinan was non-toxic for HL7702 cells but inhibited H22 cells proliferation obviously in a dose-dependent manner. *In vivo*, the proliferation of H22 hepatocarcinoma cells was inhibited by lentinan 0.4mg/kg body weight (L2, survival rate, 20%, *PPP<*0.01). Six proteins 60Sacidic ribosomal protein P2, Peroxiredoxin-2, Annexin A5, PDZ and LIM domain protein 1, Src substrate cortactin and Moesin were found as emerging proteins of the H22 cell incubated with high dose lentinan which related to cancer promotion closely. In conclusion, Thelentinan was relatively safe and could inhibit the proliferation of H22 cancer cells through immunity improvement when it's intake was in proper quantity.

## INTRODUCTION

Polysaccharides belong to a class of natural polymers that are comprised of carbohydrate monomers linked by glycosidic linkages [1]. The influence of polysaccharides’ chemical composition, molecular weight and structure on physiological functions is variable depending on their sources. Polysaccharides also possess various physicochemical properties such as gelation, solubility, low osmotic effect and surface properties which depend on their composition and chemical structure [2, 3].

Lentinan, a (1-3)-beta-d-glucan extracted from the mushroom *Lentinus edodes*, is a potent anti-cancer drug that has been licensed in Japan for anti-tumor therapy since 1985 [4–6]. Polysaccharides play important roles in the rehabilitation of patients with numerous diseases. In addition to anti-diabetic, antibiotic, antioxidant, anti-mutant, and anticoagulant activity, polysaccharides and their derivatives have shown remarkable efficacy in combating certain cancers [7]. A large number of studies have suggested that polysaccharides exhibit an anti-cancer effect following some common mechanisms: (1) prevention of oncogenesis by oral consumption of active preparations [8]; (2) enhancement of immunity against tumors directly or indirectly through combination with chemotherapy [9]; (3) inhibiting tumors such as induction of tumor cell apoptosis or inhibition of tumor metastasis [10, 11]; (4) activation of humoral and cellular immunity to prevent cancer cell proliferation [12]. Activity of immunity-enhancement is important function of lentinan, which has become a hot topic in lentinan study [13]. The mechanisms for polysaccharides enhancing immune system involve both improvement of host defense against pathogens and modulation of adaptive immunity. Regulation of the levels of lymphocytes’ proliferative activity and macrophages’ phagocytic activity, cytokines, and antibodies may contribute to lentinan's immunity-enhancement abilities [14, 15].

How to take advantage of polysaccharides such as lentinan appropriately has not been noted carefully, even if low toxicity or certain adverse effects have been reported [16]. In previous studies, the effects of cartilage polysaccharide on the apoptosis of human hepatoma BEL-7402 cells and immunoprophylaxis of murine H22 Hepatocarcinoma were reported. However, the survival status of KM mice immunized with cartilage polysaccharide was worse comparing with H22 hepatocarcinoma model mice [17].

In this study, we explored the effects of lentinan on cytotoxicity of HL7702 cells or H22 cells and mouse immunity against initial proliferation of H22 hepatocarcinoma. MTT assay was selected to test the cytotoxicity of lentinan on HL7702 cell and H22 cell *in vitro*. The cumulative survival assay, spleen lymphocyte proliferation tests and peritoneal macrophage phagocytosis assays were selected *in vivo* for the mice immunized with lentinan. Mass spectrometry (MS)-based proteomics was also used to profile the protein emerging of the H22 cell incubated with lentinan.

## RESULTS

### Cytotoxicity of lentinan *in vitro*

**Figure 1:**
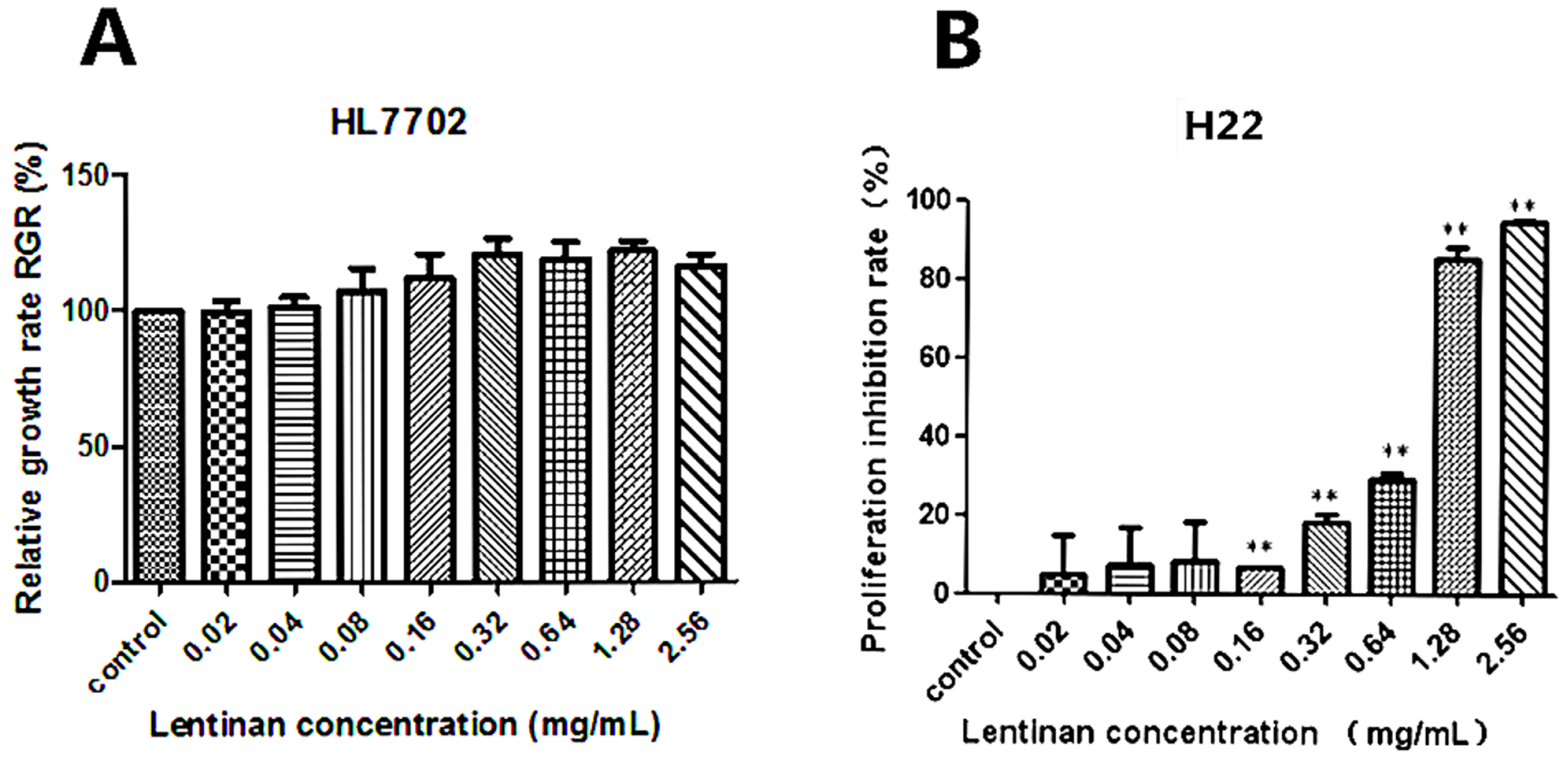
Effects of lentinan on cytotoxicity *in vitro* Human normal liver cell line HL7702 and murine liver cancer cell line H22 were treated with different concentrations of lentinan (0.02, 0.04, 0.08, 0.16, 0.32, 0.64, 1.28 and 2.56 mg/mL) for 24 h. The relative growth rate (RGR, %) of HL7702 cells and proliferation inhibition rate of H22 cells were tested. **(A)** The relative growth rate (RGR, %) of HL7702 cells treated with different concentrations of lentinan. **(B)** The proliferation inhibition rate of H22 cells treated with different concentrations of lentinan. The asterisks represent statistical significance (^*^P < 0.05 and ^**^P < 0.01) compared with the control value.

To confirm the general cytotoxicity of lentinan on HL7702 cells and H22 cells, cell proliferation condition was measured by MTT assay. For HL7702 cells, the RGR of the control cells was considered as 100%. After treatment with different concentrations of lentinan (0.02, 0.04, 0.08, 0.16, 0.32, 0.64, 1.28 and 2.56 mg/mL) for 24 h. The proliferation rate of HL7702 cells did not increased in a dose-dependent manner (Figure 1A), and there was no significantly increased compared to the control group. The viability of H22 cells after treatment with different concentrations of lentinan (0.02, 0.04, 0.08, 0.16, 0.32, 0.64, 1.28 and 2.56 mg/mL) was also measured and the average inhibition rate was 5.05%, 7.28%, 8.44%, 6.63%, 18.34%, 29.31%, 85.48 and 94.89% respectively. The inhibition rate of H22 cells increased in a dose-dependent manner (Figure 1B). And the inhibition rate of H22 cells significantly increased (*P* < 0.01) compared to the control group when they were exposed from 0.16 to 2.56 mg/mL of lentinan.

### Effects of lentinan on mice *in vivo*

**Figure 2:**
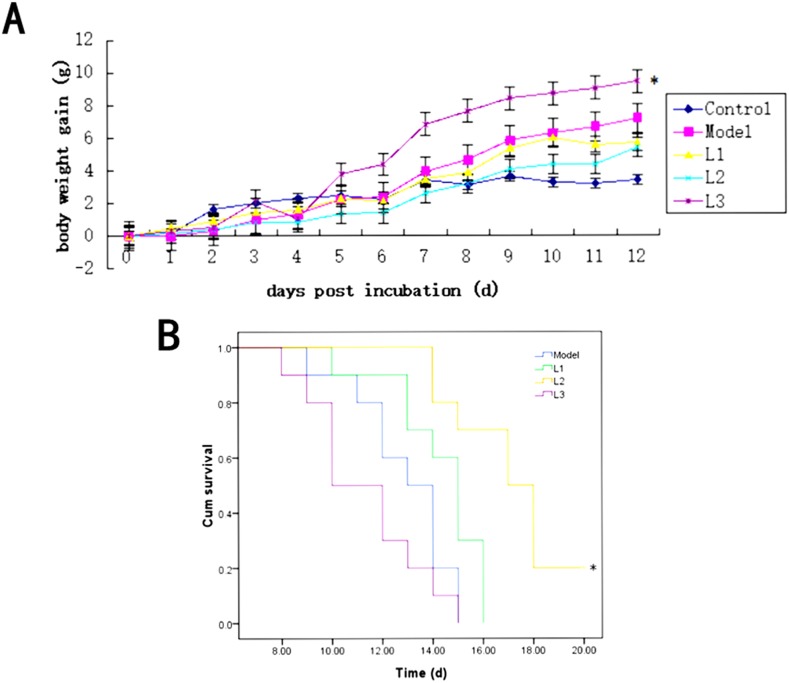
Effects of L1, L2, and L3 on mice body weight and survival L1, the concentration of lentinan was 0.02mg/kg body weight; L2, the concentration of lentinan was 0.4mg/kg body weight; and L3, the concentration of lentinan was 1mg/kg body weight. **(A)** Average body weight gain of mice in each group after modeling. **(B)** The increase in life span (ILS) or survival rate of each group. ^*^P < 0.05 compared to model.

The variation rates of the average body weight of the mice in L1 and L2 groups were between that of the control and model groups. The increase of the average mouse body weight in L3 group was higher than that of the model group (*P*<0.05). After four days, mouse body weight in L3 group showed significant increased compared to control group, while there was no significant difference in the other groups. The increase velocity of the average mouse body weight in other groups were model >L1 >L2 >control group from day 8 to 12 (Figure 2A). As shown in Figure 2B, the survival rate (20%) of the mice in L2 group was the highest among all the experimental groups, while the survival rates in other groups (L1 and L3 groups) were zero.

### Analysis of the immunoprophylaxis activity of lentinan on H22 hepatocarcinoma

**Figure 3:**
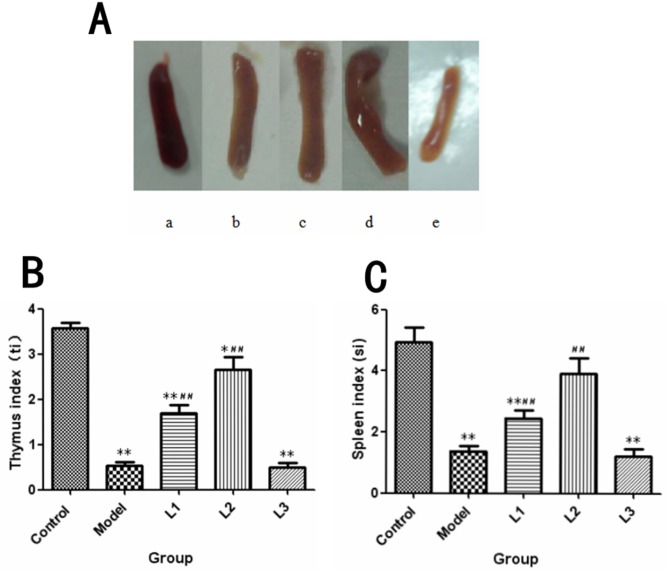
The changes in immune organs in each group L1, the concentration of lentinan was 0.02mg/kg body weight; L2, the concentration of lentinan was 0.4mg/kg body weight; or L3, the concentration of lentinan was 1mg/kg body weight. **(A)** The appearance of the spleen in each group of mice was recorded. a: control group, b: model group, c: L1 group, d: L2 group, e: L3 group. **(B)** ti (thymus index) of each group was tested. **(C)** si (spleen index) of each group was tested. ^*^P<0.05 vs control, ^**^P<0.01 vs control, ^#^*P* < 0.05 compared to model, ^##^*P* < 0.01 compared to model.

Firstly, the spleens of mice in experimental (L1, L2 and L3) and model groups appeared visibly different from that of control group. The spleens’ khaki atrophy of mice in L1, L3 and model groups had been observed visibly. However, for spleens of mice in L2 group, morphological characters were purplish red color and plump which were close to the control group (Figure 3A). The diverification of immune organ index were listed in Figure 3B/3C. The spleen (si, 1.21) and thymus (ti, 0.49) indices in L3 group showed significantly lower values than those of control group (*P*<0.01).

**Figure 4:**
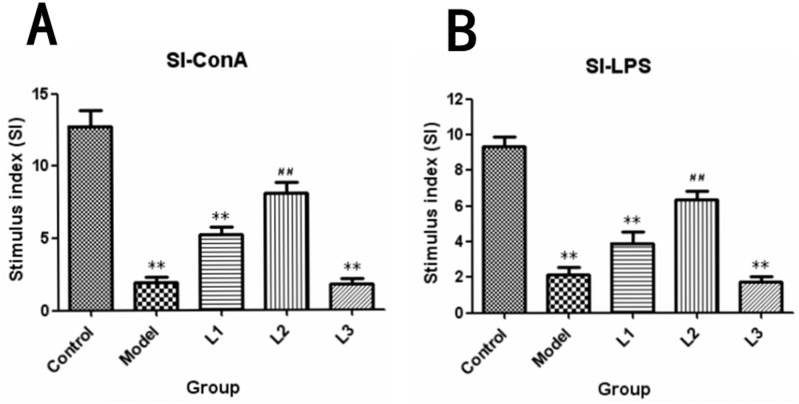
SI-stimulus index induced by ConA or LPS in lentinan groups of mice L1, the concentration of lentinan was 0.02mg/kg body weight; L2, the concentration of lentinan was 0.4mg/kg body weight; and L3, the concentration of lentinan was 1mg/kg body weight. **(A)** Stimulus index (SI)-ConA. **(B)** Stimulus index (SI)-LPS. ^**^P < 0.01 compared to control, ^##^*P* < 0.01 compared to model.

The SI (stimulation index) of each group was shown in Figure 4. It was showed that the proliferation rate of spleen lymphocytes in L3 group (SI-ConA, 1.8; SI-LPS, 1.7) was remarkably depressed compared to that of control group (*P<*0.01). While for the mice L2 group, which was treated with 0.4mg/kg body weight of soluble lentinan, showed a significant improvement in both T-cell (SI-ConA, 8.1) and B-cell (SI-ConA, 6.37) proliferation rate compared to that of model group (*P*<0.01).

**Figure 5:**
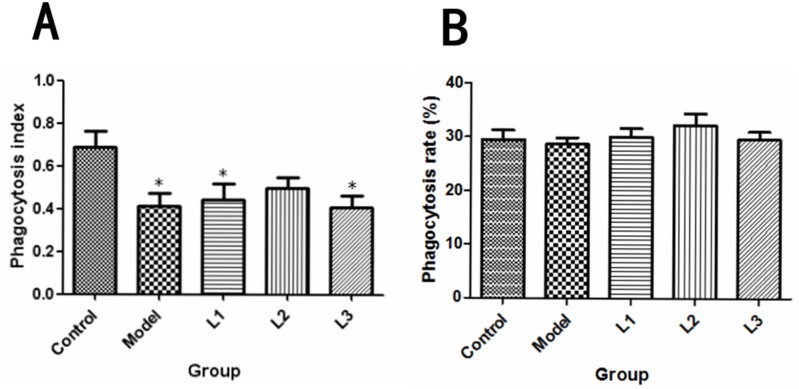
Effects of lentinan on mice peritoneal macrophage phagocytosis L1, the concentration of lentinan was 0.02mg/kg body weight; L2, the concentration of lentinan was 0.4mg/kg body weight; and L3, the concentration of lentinan was 1mg/kg body weight. **(A)** Macrophage phagocytosis rate of each group (control, model, L1, L2, and L3) was calculated. **(B)** Macrophage phagocytosis index of each group (control, model, L1, L2, and L3) was tested. ^*^P<0.05 vs control group.

As shown in Figure 5, the macrophage phagocytic indexs in L1 (phagocytic indexs, 0.44) and L3 (phagocytic indexs, 0.41) groups were significantly lower than that of control group (*P*<0.05), while there was no significant difference in phagocytosis rate (*P*>0.05) between experimental (lentinan) groups and control or model groups.

### MS analysis of emerging protein

MS/MS analysis and protein database searching were carried out to investigate the change of protein after the treatment of lentinan. Mass spectrometry analysis of H22 cells (incubated without lentinan) protein as a control versus H22 cells (incubated with lentinan) protein. In total 309 proteins were identified in H22 cells, whereas 96 new emerging proteins were detected in the H22 cells incubated with lentinan. According to the protein functional studies we focused on 6 proteins which could promote cancer occurrence or cancer cell proliferation. The 6 proteins were 60Sacidic ribosomal protein P2, Peroxiredoxin-2, Annexin A5, PDZ and LIM domain protein 1, Src substrate cortactin and Moesin as shown in Table 1.

**Table 1:** Liquid chromatography-tandem mass spectrometry (LC-MS/MS) analysis of the protein samples

Accession	Protein name	Sum PEP score	Coverage	# Peptides	# PSMs	# Unique peptides	# AAs	MW [kDa]	Calc. pI	Area
P99027	60Sacidic ribosomal protein P2	6.465735225	21.73913043	2	2	2	115	11.644	4.54	5.30E+07
Q61171	Peroxiredoxin-2	5.603677658	22.72727273	3	3	3	198	21.765	5.41	8.70E+07
P48036	Annexin A5	5.518135923	7.836990596	2	2	2	319	35.73	4.96	1.50E+07
O70400	PDZ and LIM domain protein 1	5.306689611	6.727828746	2	2	2	327	35.752	6.84	2.70E+07
Q60598	Src substrate cortactin	4.602854608	5.860805861	2	2	2	546	61.212	5.4	5.30E+07
P26041	Moesin	4.69751607	4.679376083	3	3	1	577	67.725	6.6	1.50E+07

## DISCUSSION

Searching for novel effective anticancer agents with less toxic effects is a hot topic for scientists to solve the problems in cancer therapy. Lentinus edodes, with a long history as a food source, have been believed to have health benefits including anti-tumour [18, 19], immuno-modulatory [20] and anti-microbial effects [21]. Lentinan, a polysaccharide extracted from lentinus edodes, is the best known and most potent mushroom-derived substance with anti-tumor and immuno-modulating properties [20, 22].

In the study, the cytotoxicity of lentinan over intake was tested by MTT assay *in vitro* with human normal liver cell line HL7702 and murine liver cancer cell line H22. The results revealed that lentinan was a relatively safe agent at concentration from 0.001mg/mL to 2mg/mL. For antitumor effect of lentinan *in vitro*, the proliferation rates of H22 cells treated with lentinan (0.16, 0.32, 0.64, 1.28 and 2.56 mg/mL) were inhibited obviously in a dose-dependent manner. Then the side-effect of lentinan on cancer immune especially on cancer cells clear in time should be tested *in vivo*.

In order to study the effect of lentinan on murine H22 hepatocarcinoma immunoprophylaxis, we immunized mice with lentinan. The dose conversion coefficients of per kilogram body weight from mice (20g) to human (70kg) was 9.01. The lentinan at concentration of 0.02mg/kg, 0.4mg/kg and 1mg/kg body weight were selected respectively to immunized the mice which were establish a mice model bearing with H22 hepatocarcinoma. Then We examined the physiological mechanisms activated by polysaccharides for the immunized mice.

The increase of mice body weight indicated the proliferation of ascitic tumor cells. In this study, the increase of mice body weight in L1 and L2 groups was less than that of model group, which suggested that the proliferation of H22 hepatocarcinoma cells could be inhibited to some extent treated by 0.02mg/kg or 0.4mg/kg body weight of lentinan. However, we were also puzzled by the phenomenon that the body weight increase in L3 group was significantly higher than that of control group from the fourth day to the twelfth day, which may indicate the proliferation of cancer cells could be promoted by 1mg/kg body weight of lentinan (L3 group). The survival rate of the mice in L2 group reached 20% which was higher than that of model group significantly (*P* < 0.05).

Thymus and spleen are important immune organs for vertebrates. To a certain extent, the immune condition of the body can be checked by organ appearance or an organ weight index. The growth index of the spleen (spleen weight normalized to body weight, SI), which indicates the immune function changes of the spleen [23]. The appearance or organ weight index of the spleen in L2 group was different from that of model group. The spleen and thymus indexes in L3 group were significantly lower than that of model group, indicating that treatment by high dose of lentinan (L3) before immunization could accelerate the proliferative rates of cancer cells by decrease of the immune organ functions.

The results showed that immunization with lentinan could contribute to the proliferation of both T and B lymphocytes of the mice in L2 group, nevertheless this effect was dose-independent (Figure 3). Furthermore, the proliferation of T and B lymphocytes in L3 group was lower than that of control group (*P*<0.01). The proliferation of T and B lymphocytes is an immune parameter in the investigations of lymphocyte responsiveness considering its considerable sensitivity. Cellular proliferation induced by ConA is commonly used to detect T lymphocyte immunity, and LPS-induced activation of B cells indicates B lymphocyte immunity [24, 25]. Previous reports also indicated that lentinan could promote both humoral and cellular immune responses against ovarian cancer, gastric cancer, colorectal cancer, and liver cancer [22, 24, 26, 27].

In the present study, the phagocytic index of the immunized mice in L1 or L3 group was decreased compared with control group but not model group. There were no significant difference in the phagocytic rate among control group, model group and immunized groups. Peritoneal macrophages are important for cancer immunity, because they can kill cancer cells directly or indirectly [28]. Therefore, we speculated that stimulation of macrophages may be one of the most critical mechanisms for the cancer inhibitory activity exerted by polysaccharides.

Using MS analysis, six proteins existed in H22 cells incubated with lentinan were the targets we focus on. These 6 proteins 60Sacidic ribosomal protein P2, Peroxiredoxin-2, Annexin A5, PDZ and LIM domain protein 1, Src substrate cortactin and Moesin which had relationship with cancer proliferation *in vitro* or *in vivo* according to the previous reports. 1) The phosphorylation level of P2 protein is one of the regulatory mechanisms for the overall rate of protein elongation. Thus, increased expression of P2 protein can increasing proliferation rate of cancer cells [29–31]. 2) Peroxiredoxin 2 is a member of the peroxiredoxin family, which has been found to be elevated in several human cancer cells and tissues, including colorectal cancer, pancreatic cancer and breast cancer, and it influences cells’ survival, proliferation, and apoptosis processes, which suggests a possible role for Peroxiredoxin 2 in the maintenance of cancer cell[32–34]. 3) Annexin A5 a calcium-binding protein which is involved in membrane organization and dynamics has been implicated in the carcinogenesis of several carcinomas. The biological function of endogenous annexin A5 was its possible influence on proliferation and invasion capacity [35, 36]. 4) PDZ and LIM domain protein 1 (PDLIM1) also known as CLP36, Elfin or CLIM1 is critical for promoting breast cancercell migration and invasion *in vitro* and metastasis *in vivo*, whereas it is dispensable for breast cell proliferation and anchorage-independent growth *in vitro* and tumor growth *in vivo* [37]. 5) Cortactin frequently over expressed in cancer was first identified as one of the major substrates for src kinase [38]. Promotion of tumor invasion and metastasis was the role of cortactin over expression *in vivo*. Many other studies suggest that cortactin promotes cell motility and invasion, including a critical role in invadopodia, actin rich-subcellular protrusions associated with degradation of the extracellular matrix by cancer cells [39]. 6) Moesin which plays a key role in the control of cell morphology, motility, adhesion and other processes of tumourigenesis is a linker between the actin cytoskeleton and the plasma membrane [40]. The study of Wu M, et al. has showed the expression of moesin was strongly negatively correlated with the patient progression-free survival and overall survival. And the moesin protein involved in the genesis and progression of astrocytomas and might be regarded as an independent predictor of poor prognosis [41].

## MATERIALS AND METHODS

### Animals

SPF female KM mice weighted 18-22 g were purchased from the animal center of Hebei Medical University (SCXK (JI) 2013-1-003, 1410014, Shijiazhuang, China). All experiments were conducted following the Guide for the Care and Use of Laboratory Animals of National Institutes of Health. This study was approved by the animal ethics committee of Hebei University of Science and Technology(Permission number: 2014-A02-01). The animals were maintained in an environment with controlled temperature (20 to 25 °C) and humidity (50% ± 5%), with food and water available at any time and a natural light. The health of the mice was monitored every day. Before blood collection or immune organ index test, mice were anesthetized with ether, and the other mice were euthanized by cervical dislocation under anesthesia with isoflurane.

### Cell lines

The human normal liver cell line HL7702 cells and murine liver cancer cell line H22 cells (China PLA General Hospital, Beijing, China) were cultured in RPMI 1640 medium supplemented with 10% heat-inactivated fetal bovine serum (FBS) and antibiotics (100U/ml penicillin and 100 g/ml streptomycin) at 37°C in an atmosphere of 5% CO_2_.

### Lentinan solution

Lentinan used in this study was gifted from Kang Yuan (Jiangsu, China). Different concentrations of lentinan saline solution were prepared, 0.02 mg/mL, 0.04 mg/mL, 0.08 mg/mL, 0.16 mg/mL, 0.32 mg/mL, 0.64 mg/mL, 1.28 mg/mL, 2.56 mg/mL and 0.02mg/kg body weight (L1group), 0.4mg/kg body weight (L2 group), 1mg/kg body weight (L3 group) were used for the tests *in vitro* and *in vivo* respectively.

### Cytotoxicity assay *in vitro*

The cytotoxicity of lentinan on HL7702 and H22 cells *in vitro* was tested by 3-(4,5-dimethylthiazol-2-yl)-2,5-diphenyl tetrazolium bromide (MTT) assay. HL7702 and H22 cells at logarithmic phase were seeded in 96-well micro-plates (Corning, NY, USA) at a density of 5×10^3^ and 1×10^6^ cells per well per 100 μL of medium respectively. After incubation for 24 h, the cells were treated with various concentrations of lentinan solution (0.02-2.56 mg/mL) and continued to be incubated at 37°C for another 24h. Then the cells seeded in 96-well plates were harvested and pre-incubated with 0.5 mg/ml MTT (Solarbio, Beijing, China) for 4 h at 37 °C. When MTT incubation was completed, supernatant was discarded. Then, 150 μL of dimethyl sulfoxide (DMSO, Solarbio, Beijing, China) was added to each well and incubated for 10 min with shaking to dissolve the crystallization. Finally, the absorbance of each well was measured at 570 nm using a microplate reader (Model 680, Bio-Rad, Hercules, Calif., U.S.A.). RPMI-1640 medium was used to determine the background (blank) OD, which was subtracted from the OD of the samples. The relative growth rate (RGR, %) (n=3) of HL7702 cell was: OD_treatment_ / OD_control_×100%. The inhibition rate for H22 cell proliferation (%) (n=3) was : (OD_control_–OD_treatment_) / OD_control_×100%.

### Establishment of mouse model bearing H22 tumors

A total of 100 KM mice were randomly divided into a control group, model group, lentinan 1(L1) group, lentinan 2 (L2) group, and lentinan 3 (L3) group,with 20 mice in each group. The lentinan groups were immunized (intraperitonially [i.p.]) with lentinan saline solution (L1: 0.02mg/kg body weight ; L2: 0.4mg/kg body weight; 1mg/kg body weight) once a week for 3 weeks. The control group was treated with 0.9% normal saline. The model group was used to be as positive group and treated with 0.9% normal saline. One week after the third immunization, all of the mice except the control group were injected i.p. with H22 hepatocarcinoma cells (1.5×10^6^ cells/mouse) to establish the H22 hepatocarcinoma mouse model. After establishment of the H22 mouse model, the body weight (BW) of the mice in each group was measured and recorded every day for 12 days. Ten mice in each group were selected randomly to calculate the immune organ (thymus and spleen) index or cellular immunity (T-cell proliferation test and peritoneal macrophage phagocytosis) on the twelfth day after establishment of the H22 mouse model. The spleens or thymus were collected from each mouse, and the growth index for the thymus and spleen were calculated and recorded.

Growth index of spleen = spleen weights (mg) / BW (g)

Growth index of thymus = thymus weights (mg) / BW (g)

Twenty days after establishment of the H22 mouse model, the cum survival of the mice in each group were tested with Kaplan-Meier method (n=10).

### Spleen lymphocyte proliferation tests

For the lymphocyte proliferation assay, spleen lymphocytecells from each group were collected and suspended at concentration of 2×10^6^ viable cells/mL in RPMI 1640 (containing 10% FBS) medium. Then Concanavalin A (ConA) or Lipopolysaccharide (LPS) (10 μg/mL) were mixed with spleen cell suspension at a ratio of 3:1 and added in 96-well plates, 100 μL/well. The cell cultures were incubated at 37 °C for 72 h. Four h before the cells were harvested, 0.5 mg/mL of MTT solution was added. Then 150 μL of DMSO was added to each well and incubated for 10 min with shaking to dissolve the crystallization. Finally, the absorbance of each well was read at 570 nm using a microplate reader.

Stimulus index (SI)= OD_experimental_ / OD_control_

### Peritoneal macrophage phagocytosis assays

In order to test the phagocytosis activity of peritoneal macrophages, mice in each group was injected (i.p.) with bouillon culture medium (Amidulin, 6%) once a day. Three days later, Chick erythrocytes (1% suspension) was injected (i.p.) into mice and the specimens of peritoneal fluid was harvested 0.5 h later. Then the peritoneal fluid of mice in each group was smeared on glass slides and stained with Wright Giemsa. 100 macrophages of each sample were observed. When chick erythrocytes appeared in the cytoplasm, the macrophages cells were considered to be actively phagocytic. The number of the activated macrophages and the number of chick erythrocytes in macrophages were recorded. The phagocytosis rate and phagocytosis index were calculated according to the reference [12].

Phagocytosis index= number of the phagocytic chick erythrocyte/100

Phagocytosis rate (%) = (number of the activated macrophages/100) × 100%

### LC-MS/MS analysis and protein database searching for emerging protein

H22 cells at logarithmic phase were incubated with or without lentinan (1.28mg/mL) at 37°C for 24h. The co-culture was frozen, thawed 5 times to inactivate living H22 hepatocarcinoma cells with PMSF and the supernatant was selected as samples. The protein sample was further concentrated using Amicon Ultra-15 centrifugal filter MWCO3000 (Millipore). Protein concentration of the samples was measured by Bradford Assay (Solarbio, Bradford Protein Assay Kit, Beijing, China). An aliquot of the concentrated preparation containing 100 μg protein was diluted with 50 mM NH_4_HCO_3_ to 70μL and added DTT with final concentration 10mM at 56°C for 1h. The sample was then incubated with iodoacetamide (10mM final concentration) at room temperature for 40 minutes before digestion with trypsin (trypsin:protein 1:50 w/w) at 37 °C for 14 h. After speed-vac, the sample peptides were reconstituted in 50 mM NH_4_HCO_3_ for LC-MS analysis.

Liquid chromatograohy/mass spectrometry was performed on Thermo Scientific™ Q Exactive equipped with a Nanospray Floex Ion-Soure. Peptides suspension dissolved in 0.1% formic acid were separated by nano-high performance liquid chromatography (Eksigent Technologies) on a secondary reversed-phase analytical column (Eksigent, C18, 3μm, 150 mm^*^75μm). Buffer A for the pump consisted of 0.1% formic acid in LC/MS-grade water; buffer B for the pump consisted of 0.1% formic acid in LC/MS-grade acetonitrile. Gradient conditions for pump B were as follows: 0% to 35% B from 0 to 90 minutes. A total of 5 mL of the prepared peptides was injected onto the trap column for concentration/purification. Flow rates were 300 nL/min. Some parameters in Orbitrap were as follows: spray voltage, 2.0 kV; capillary temperature, 250°C; m/z (mass to charge ratio) range (ms), 350 to 1800. AGC ion injection targets for each FTMS scan were 70,000 (100 ms max injection time). AGC ion injection targets for each MS2 scan were 17,500 (50 ms max ion injection time). Full MS/dd-MS2 (Top10) was used in this analysis, with a dynamic exclusion time of 25 s. Both identification and quantification were done by Proteome Discoverer (version 2.1demo, Thermo Fischer Scientific). Uniprot-mouse database was used for data mining. Peptides were generated from a tryptic digestion with up to 2 missed cleavages, dynamic modifications of methionine oxidation, and a static modification of cysteine carbamidomethylation. Precursor mass tolerance was 10 ppm, and product ions were searched at 0.02-Da tolerances. Peptide spectral matches were validated using percolator based on q values at a 1% false discovery rate.

### Statistical analysis

The data were analyzed using SPSS software (version 11.5, SPSS, New York, NY, USA) and *P* < 0.05 was considered as statistically significant. All the data were presented as mean±SD.

## CONCLUSION

In conclusion, the safety of lentinan was relative. Lentinan (from 0.16 mg/mL to 2.56 mg/mL) was non-toxic for normal liver HL7702 cells, and the H22 mice liver cancer cells proliferation were inhibited by lentinan dramatic *in vitro*. On the other hand, lentinan could promote the cancer cell proliferation at concentration of greater than 1mg/kg body weight *in vivo*. The cellular immune functions of H22 model mice were improved in L2 group (0.4 mg/kg body weight lentinan), mainly reflected in inhibition of cancer cell proliferation and protection of immune organs. Therefore, L2 may assist in liver cancer immunoprophylaxis. Conversely, L3 (1mg/kg body weight lentinan) not only did not inhibit cancer cell proliferation, but it promoted the proliferation of cancer cells. 60Sacidic ribosomal protein P2, Peroxiredoxin-2, Annexin A5, PDZ and LIM domain protein 1, Src substrate cortactin and Moesin were detected by MS assay. Based on the previous studies, these six proteins may play an important role in the promotion of cancer cell proliferation in this study. Thus, we need to be attentive to the dose of lentinan used in clinics based on these study results. As a double-edged sword, lentinan may make important contributions to cancer therapy or immunoprophylaxis when we used properly.
